# A novel role of the aryl hydrocarbon receptor (AhR) in centrosome amplification - implications for chemoprevention

**DOI:** 10.1186/1476-4598-9-153

**Published:** 2010-06-17

**Authors:** Nina Korzeniewski, Sarah Wheeler, Payel Chatterjee, Anette Duensing, Stefan Duensing

**Affiliations:** 1 Cancer Virology Program, University of Pittsburgh Cancer Institute, Pittsburgh, PA 15213, USA; 2 Molecular Virology and Microbiology Graduate Program, University of Pittsburgh School of Medicine, Pittsburgh, PA 15261, USA; 3 Cellular and Molecular Pathology Graduate Program, University of Pittsburgh School of Medicine, Pittsburgh, PA 15261, USA; 4 Department of Pathology, University of Pittsburgh School of Medicine, Pittsburgh, PA 15261, USA; 5 Department of Microbiology and Molecular Genetics, University of Pittsburgh School of Medicine, Pittsburgh, PA 15219, USA

## Abstract

**Background:**

Centrosome aberrations can cause genomic instability and correlate with malignant progression in common human malignancies such as breast and prostate cancer. Deregulation of cyclin/cyclin-dependent kinase 2 (CDK2) activity has previously been shown to be critically involved in centrosome overduplication. We therefore test here whether small molecule CDK inhibitors derived from the *bis*-indole indirubin can be used to suppress centrosome aberrations as a novel approach to chemoprevention of malignant progression.

**Results:**

As expected, we found that the CDK inhibitor indirubin-3'-oxime (IO) suppresses centrosome amplification in breast cancer cells. However, we made the unexpected discovery that indirubin-derived compounds that have been chemically modified to be inactive as kinase inhibitors such as 1-methyl-indirubin-3'-oxime (MeIO) still significantly reduced centrosome amplification. All indirubins used in the present study are potent agonists of the aryl hydrocarbon receptor (AhR), which is known for its important role in the cellular metabolism of xenobiotics. To corroborate our results, we first show that the coincidence of nuclear AhR overexpression, reflecting a constitutive activation, and numerical centrosome aberrations correlates significantly with malignancy in mammary tissue specimens. Remarkably, a considerable proportion (72.7%) of benign mammary tissue samples scored also positive for nuclear AhR overexpression. We furthermore provide evidence that continued expression of endogenous AhR is critical to promote centriole overduplication induced by cyclin E and that AhR and cyclin E may function in the same pathway. Overexpression of the AhR in the absence of exogenous ligands was found to rapidly disrupt centriole duplication control. Nonetheless, the AhR agonists IO and MeIO were still found to significantly reduce centriole overduplication stimulated by ectopic AhR expression.

**Conclusions:**

Our results indicate that continued expression of endogenous AhR promotes centrosome amplification in breast cancer cells in a pathway that involves cyclin E. AhR agonists such as indirubins inhibit centrosome amplification even when stimulated by ectopic expression of the AhR suggesting that these compounds are potentially useful for the chemoprevention of centrosome-mediated cell division errors and malignant progression in neoplasms in which the AhR is overexpressed. Future studies are warranted to determine whether individuals in which nuclear AhR overexpression is detected in benign mammary tissue are at a higher risk for developing pre-cancerous or cancerous breast lesions.

## Background

The aryl hydrocarbon receptor (AhR) is a cytoplasmic, ligand-activated transcription factor that mediates the toxicity of halogenated or polycyclic aromatic hydrocarbons including dioxins or benzo[a]pyrene [1]. Long-term exposure to such xenobiotics has been implicated in an increased risk for common human malignancies including breast and prostate cancer [2,3]. Both cancer types frequently show an aberrant AhR expression [2,4], however, there is compelling evidence that the AhR can promote cancer formation independent of the presence of exogenous ligands [2,5,6]. Support for this notion stems mainly from studies in breast cancer.

The AhR was found to be overexpressed in primary breast cancers and mammary tumor cell lines in the absence of detectable xenobiotics [2] and there is convincing evidence for a critical role of endogenous AhR in proliferation control in tumor cells [1]. Inhibition of the AhR was found to be associated with slow growth and downregulation of cyclin and CDK2 expression [7] suggesting that its continued expression is important for cell cycle progression [1]. The tumorigenic function of the AhR is underscored by the fact that constitutively active AhR induces stomach tumors in rodents [8]. Paradoxically, acute activation of the AhR by 2,3,7,8-tetrachlorodibenzo-*p*-dioxin (TCDD; dioxin) inhibits tumor cell proliferation through mechanisms that involve upregulation of the CDK inhibitor p27^Kip1^, binding of the pRB tumor suppressor, suppression of E2F-mediated transcription as well as inhibition of hormone signaling [9-11]. Taken together, these results highlight that altered expression of endogenous AhR that is not activated by exogenous ligand has, in general, pro-proliferative and tumor-promoting properties, whereas exogenously activated AhR can have anti-proliferative activities.

Disruption of cell cycle control and increased proliferation is a common finding in breast cancer [12]. A number of studies have shown that aberrant cell proliferation promotes not only the production of increased numbers of daughter cells but at the same time increases the risk of genomic instability, another hallmark of most epithelial malignancies [13,14]. A link between deregulated cell cycle control and genomic instability is provided by the centrosome duplication cycle [15]. Centrosomes function as major microtubule organizing centers in most mammalian cells during interphase and mitosis [16]. Tumor cells frequently show abnormal centrosome numbers that can increase the risk for cell division errors, chromosome missegregation and aneuploidy [17,18]. Besides promoting polarity disturbances, it has recently been reported that extra centrosome can lead to merotelic microtubule attachment to kinetochores thereby causing chromosome segregation defects [19]. In breast cancer, centrosome aberrations have been detected in pre-invasive ductal lesions and independently of inactivation of p53 [20-22]. The latter finding is critical because it underscores that centrosome aberrations may directly cause cell division errors in breast cancer and are not merely a consequence of genomic instability associated with p53 loss or unrelated cellular defects [23,24]. It furthermore suggests that centrosome aberrations may directly arise from disruption of centrosome duplication control. A number of genes frequently altered in breast cancer including BRCA1 [25] as well as estrogen signaling [26,27] have been implicated in centrosome amplification.

Given the fact that centrosome aberrations arise early during malignant progression as shown by Lingle and co-workers [20] and have a potentially detrimental impact on genome integrity, it has been proposed that the centrosome duplication process may be a target to prevent progressive chromosomal instability in early stage lesions and hence progression to invasive cancer [28].

Deregulation of cyclin E/CDK2, an early and frequent finding in breast cancer [29], has recently been shown to cause an aberrant recruitment of the centrosomal protein kinase PLK4 to centrioles thereby promoting centriole and centrosome overduplication [30]. Given the crucial role of CDK2 in centriole amplification and the high frequency of its deregulation in early stage breast cancer [12,29], we asked here whether targeting CDK2 can prevent centrosome aberrations in breast cancer cells.

Using the indirubin-derived CDK inhibitor indirubin-3'-oxime (IO) and its counterpart 1-methyl-indirubin-3'-oxime (MeIO), which has been chemically modified to be inactive as a kinase inhibitor [11], we unexpectedly discovered that both kinase-active and kinase-inactive compounds effectively inhibited centrosome overduplication in breast cancer cell lines. Importantly, IO, MeIO and a number of other indirubins used in our experiments are potent AhR agonists [31-33]. In an effort to further elucidate a connection between AhR and centrosome aberrations, we found that the combined presence of nuclear AhR overexpression and numerical centrosome aberrations correlated significantly with malignancy in mammary tissue specimens. Surprisingly, we found that a considerable proportion of benign mammary tissue samples scored positive for nuclear AhR overexpression. *In vitro *experiments showed that continued expression of endogenous AhR is critical to promote centriole overduplication induced by cyclin E and that the AhR and cyclin E may function in the same pathway. Furthermore, overexpression of the AhR in the absence of exogenous ligand was found to rapidly disrupt centriole duplication control. Nonetheless, AhR agonists including IO and MeIO were still found to significantly reduce centriole overduplication stimulated by ectopic AhR expression.

Collectively, our results provide evidence for a novel role of the AhR in centriole duplication and suggest that AhR-agonistic indirubins are potentially useful to prevent centrosome-mediated chromosome instability and malignant progression even in lesions that overexpress the AhR. Whether individuals with nuclear AhR overexpression are at a higher risk for the formation of pre-cancerous and cancerous lesions will be the subject of future studies.

## Methods

### Cell culture, transfections and inhibitor treatments

Human HCC1806 and MCF-7 mammary tumor cell lines were obtained from ATCC and maintained as recommended by the vendor. MCF-7 cells were stably transfected with either pCMV-based plasmids encoding cyclin E (kindly provided by Philip Hinds, Tufts University, Boston, MA) [34] or empty vector (neo) or pEGFP-centrin-1-GFP (kindly provided by Michel Bornens, Institut Curie, Paris, France) [35]. For transient overexpression, a pcDNA-based human AhR plasmid (kindly provided by David Sherr, Boston University, Boston, MA) [36] was used. Indirubin-3'-oxime (IO), 1-methyl-indirubin-3'-oxime (MeIO), 6-bromo-indirubin-3'-oxime (BIO), 1-methyl-6-bromo-indirubin-3'-oxime (MeBIO) and 6-bromo-indirubin-3'-acetoxime (BIA) (all compounds were generously provided by Laurent Meijer, Station Biologique, Roscoff, France) were dissolved in DMSO and used at a 1 μM concentration for 24 h. DMSO was included as solvent control in all experiments.

### Immunological methods

Immunoblot analyses of whole cell protein extracts were performed as previously described [37]. For immunofluorescence microscopic analysis of AhR expression and centrosome numbers, a breast multitissue array (BR1003; Biomax US) was used. Sections were processed as previously described [38] and incubated with an anti-AhR antibody (Santa Cruz) at a 1:100 dilution or an anti-γ-tubulin antibody (Sigma) at a 1:500 dilution for at least two days at 4°C followed by FITC-conjugated secondary antibodies (Jackson Immunoresearch). Immunofluorescence staining for centrin (antibody kindly provided by Jeffrey Salisbury, Mayo Clinic, Rochester, MN) was performed as previously described [37]. Nuclei were stained with DAPI. Sections were analyzed using an Olympus AX70 epifluorescence microscope. For quantification of AhR staining, tissue cores were scored positive when at least a single breast epithelial or tumor cell with nuclear AhR expression was detected (see Additional File 1). For quantification of centrosome abnormalities, three sections of the same multi-tissue array were stained independently for γ-tubulin and the proportion of cells with centrosome aberrations (more than two centrosome per cell) was quantified (see Additional File 1). A core that contained at least one cell with abnormal centrosome numbers in one of the three independent experiments scored as positive. All tissue cores with insufficient staining quality were excluded from our analysis ("not assessable", NA in Additional File 1).

### Small-interfering RNA (siRNA)

Synthetic RNA duplexes to reduce AhR expression and control RNA duplexes were obtained commercially (Ambion) and used according to manufacturer's protocol.

### Statistical Analysis

Student's two-tailed t test for independent samples was used wherever applicable. To assess the correlation between nuclear AhR overexpression, numerical centrosome abnormalities or a combination of both, respectively, with malignancy, benign (normal and hyperplasia) and malignant (dysplasia and cancer) samples were scored as either positive or negative for each or both markers (columns two and six of Additional File 1). See Immunological Methods for more details of the scoring method. 2 × 2 contingency tables were created followed by a two-tailed Fisher exact probability test.

## Results

### Indirubins inhibit centriole overduplication in breast cancer cells

Given the crucial role of cyclin/CDK2 complexes in promoting centriole overduplication [39], we sought to determine whether indirubin-derived small molecule CDK inhibitors can suppress centrosome amplification in breast cancer cells.

**Figure 1:**
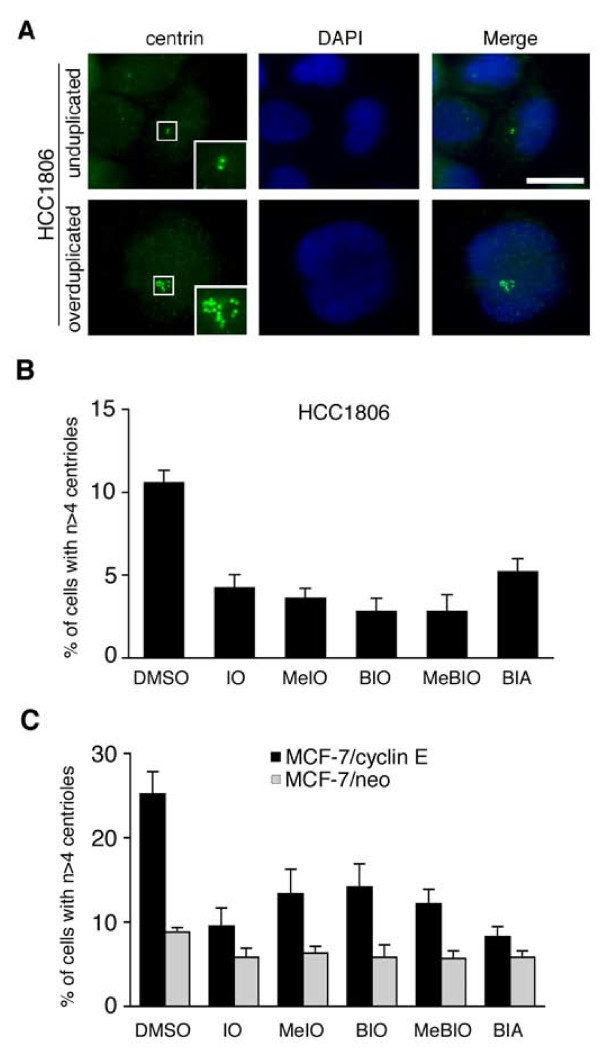
**Indirubins inhibit centriole overduplication in breast cancer cell lines**. (A) Immunofluorescence microscopic analysis of HCC1806 breast cancer cells for centrin to visualize normal centrioles (top panels) in contrast to centriole overduplication (bottom panels). Nuclei stained with DAPI. Scale bar indicates 10 μm. (B, C) Quantification of the proportion of HCC1806 or MCF-7 cells manipulated to stably express empty vector (MCF-7/neo) or cyclin E (MCF-7/cyclin E) with aberrant centriole numbers (>4 per cell) after treatment with 0.1% DMSO as solvent control or 1 μM indirubin-3'-oxime (IO), 1 μM 1-methyl-indirubin-3'-oxime (MeIO), 1 μM 6-bromo-indirubin-3'-oxime (BIO), 1 μM 1-methyl-6-bromo-indirubin-3'-oxime (MeBIO) or 1 μM 6-bromo-indirubin-3'-acetoxime (BIA) for 24 h. Each bar represents mean and standard error of at least three independent experiments with a minimum of 50 cells counted per experiment. Only mononucleated cells were assessed for centriole aberrations to exclude polyploid or otherwise altered cells.

Triple-negative (estrogen receptor-, progesterone receptor, HER2-negative) HCC1806 breast cancer cells were treated with indirubin-3'-oxime (IO) in comparison to kinase-inactive 1-methyl-indirubin-3'-oxime (MeIO) and several indirubin analogues (Table 1), all at a 1 μM concentration for 24 h, and stained for centrin to visualize individual centrioles (Fig. 1A). In all of our experiments, bi- or multinucleated cells were excluded from the analysis since they are commonly associated with centriole accumulation in contrast to centriole overduplication [40]. As expected [28], IO, which effectively inhibits CDK2, CDK1 and GSK-3β [41], was found to lead to a significant 2.5-fold reduction of the proportion of cells with abnormal centrosome numbers from 10.6% in DMSO-treated controls to 4.2% in cells treated with IO (p ≤ 0.0001; Fig. 1B). Surprisingly, however, the kinase-inactive counterpart of IO, MeIO, also caused a significant 2.9-fold reduction of cells with centrosome aberrations to 3.6% (p ≤ 0.0001; Fig. 1B). Similar results were obtained when we treated HCC1806 cells with 6-bromo-indirubin-3'-oxime (BIO; 2.8%, p ≤ 0.0001), the kinase-inactive compound 1-methyl-6-bromo-indirubin-3'-oxime (MeBIO; 2.8%, p ≤ 0.0001) or 6-bromo-indirubin-3'-acetoxime (BIA; 5.2%, p ≤ 0.001), which was the least effective indirubin analogue in terms of reduction of centriole overduplication (Fig. 1B).

**Table 1:** Activity spectrum of indirubins

Compound	CDK2 inhibition	CDK1 inhibition	GSK-3β inhibition	AhR activation
Indirubin-3'-oxime (IO)	++^a^	++^b^	+++^b^	++^b^
1-Methyl-indirubin-3'-oxime (MeIO)	inactive^b^	-^b^	-^b^	++^b^
6-Bromo-indirubin-3'-oxime (BIO)	++^a^	++^b^	+++^b^	++^b^
1-Methyl-6-bromo-indirubin-3'-oxime (MeBIO)	inactive^b^	-^b^	-	+++^b^
6-Bromo-indirubin-3'-acetoxime (BIA)	+^a^	-^b^	+++^b^	++^b^

IC_50 _(kinases, μM), EC_50 _(AhR, μM): <0.1 = +++; <1 = ++; 1-10,000 = +; >10,000 = -

^a^Data from Meijer et al., Chem. Biol. 2003; 10:1255-1266.

^b^Data from Knockaert et al., Oncogene 2004; 23:4400-4412.

We next manipulated MCF-7 breast cancer cells to stably overexpress cyclin E in order to hyperstimulate centriole overduplication. When MCF-7/cyclin E cells were treated with 1 μM IO or MeIO for 24 h, a significant 2.7-fold reduction of the proportion of cells with aberrant centriole numbers from 25.2% in DMSO-treated controls to 9.5% in IO-treated cells (p ≤ 0.01) and 13.3% in MeIO-treated cells (1.9-fold; p ≤ 0.05) was detected. A decrease of the proportion of cells with aberrant centriole numbers was also detected when MCF-7/cyclin E cells were treated with BIO (14.1%; p ≤ 0.05), kinase-inactive MeBIO (12.1%; p ≤ 0.005) or BIA (8.3%; p ≤ 0.0001). A moderate decrease of cells with numerical centriole aberrations was also detected in control MCF-7 cells stably expressing empty vector (MCF-7/neo) treated with IO (5.8%; p ≤ 0.05), MeIO (6.3%; p ≤ 0.05), BIO (5.8%; p > 0.05), MeBIO (5.7%; p ≤ 0.05) or BIA (5.8%; p ≤ 0.01) in comparison to DMSO (8.8%).

The reduction of cells with centriole aberrations following treatment with kinase-inactive MeIO or MeBIO was unexpected. MeIO as well as all other indirubins tested have previously been reported to exert kinase-independent activities through binding and activation of the AhR (Table 1). Both cell types used, HCC1806 and MCF-7 cells showed a robust protein expression of the AhR by immunoblot analysis (not shown). Since the AhR has not been implicated in centriole duplication control before, we sought to determine the biological relevance of this association and analyzed the expression of the AhR in correlation to centrosome abnormalities in primary breast tissue samples.

### AhR overexpression and centrosome aberrations correlate with malignancy in mammary tissue specimens

**Figure 2:**
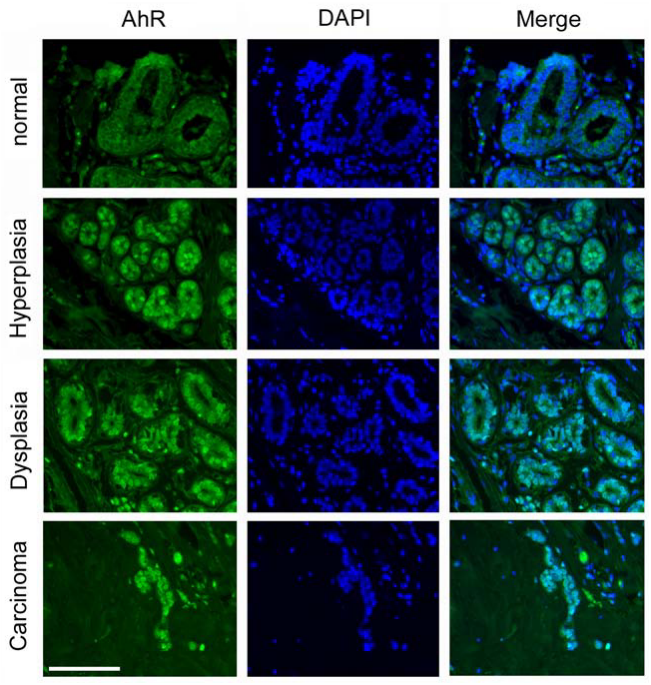
**The AhR is overexpressed in non-malignant and malignant breast tissue**. Examples of an immunofluorescence microscopic analysis of non-malignant and malignant breast tissue specimens for nuclear overexpression of the AhR. Note the nuclear staining in the hyperplastic tissue specimen (second row from the top) as well as in the dysplasia and carcinoma. Nuclei stained with DAPI. Scale bar indicates 100 μm.

Expression of the AhR was analyzed in non-malignant and malignant breast tissue specimens using a multi-tissue array. A total of six normal breast tissue samples, 16 hyperplastic tissue samples, 13 dysplastic tissue samples and 19 cancerous tissue samples were analyzed (Additional File 1). We detected nuclear overexpression of the AhR (Fig. 2) in four of six normal samples (66.7%), 12 of 16 hyperplastic samples (75%), nine of 13 dysplastic samples (69.2%) and 17 of 19 breast cancer samples (89.5%, see Methods for details of scoring procedure). These results show that the AhR is overexpressed in a subset of benign and malignant breast tissue samples and, furthermore, that its expression tends to increase with malignant progression.

**Figure 3:**
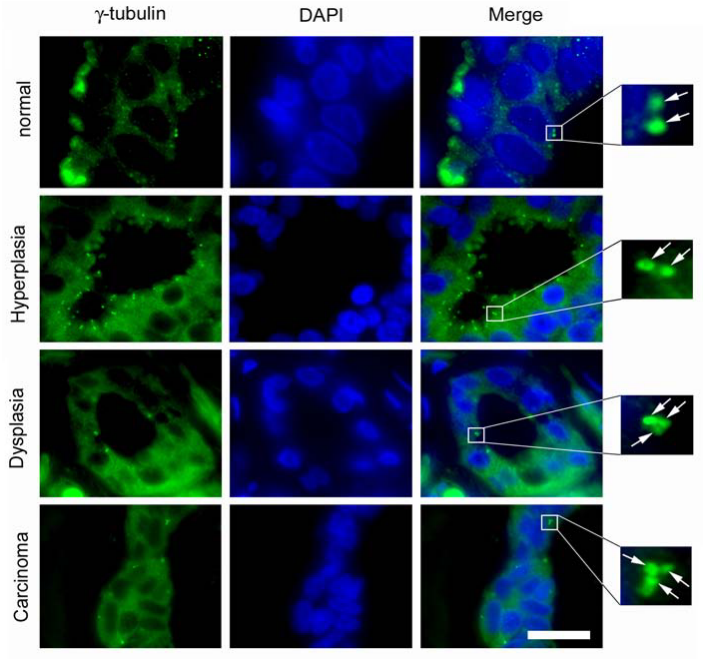
**Centrosome aberrations in breast tissue**. Examples of an immunofluorescence microscopic analysis of non-malignant and malignant breast tissue specimens for the centrosome marker γ-tubulin. Note the presence of extra centrosomes in the dysplastic and cancerous tissue specimens. Arrows in inset point to centrosomes. Nuclei stained with DAPI. Scale bar indicates 50 μm.

We then analyzed adjacent sections of the same multitissue array for the presence of supernumerary centrosomes (Fig. 3). Abnormal centrosome numbers (more than two per cell) were assessed following immunofluorescence microscopy for γ-tubulin, a marker of the pericentriolar material. Most cells with aberrant centrosome numbers contained a group of three centrosomes (see Fig. 3, bottom two panels). At least one cell with abnormal centrosome numbers was detected in two of six normal breast tissue samples (33.3%), nine of 16 hyperplastic lesions (56.3%), 12 of 13 dysplastic lesions (92.3%) and 18 of 19 cancerous lesions (94.7%). The mean percentage of cells with numerical centrosome aberrations was 0.5% in normal controls. 2.9% in hyperplastic samples, 4.6% in dysplasias and 9.6% in invasive carcinomas (see Additional File 1).

We next asked whether the presence of nuclear AhR overexpression or centrosome aberrations correlates with malignancy. Whereas we found a statistically significant correlation between numerical centrosome aberrations and malignancy (dysplasia and cancer, p ≤ 0.0005, two-tailed Fisher exact probability test), no such correlation was detected for AhR overexpression alone. However, the coincidence of nuclear AhR overexpression and abnormal centrosome aberrations in a given tissue sample was found to correlate significantly with malignancy (p ≤ 0.005, two-tailed Fisher exact probability test).

Taken together, these results suggest a positive correlation between nuclear AhR overexpression, centrosome amplification and malignancy in mammary tissue samples. They also provide evidence for a surprisingly high level of nuclear AhR overexpression in normal and hyperplastic breast tissue specimens and hence support the notion that the AhR may play a role in early steps of breast carcinogenesis.

### AhR is necessary to promote centriole overduplication in breast cancer cell lines

**Figure 4:**
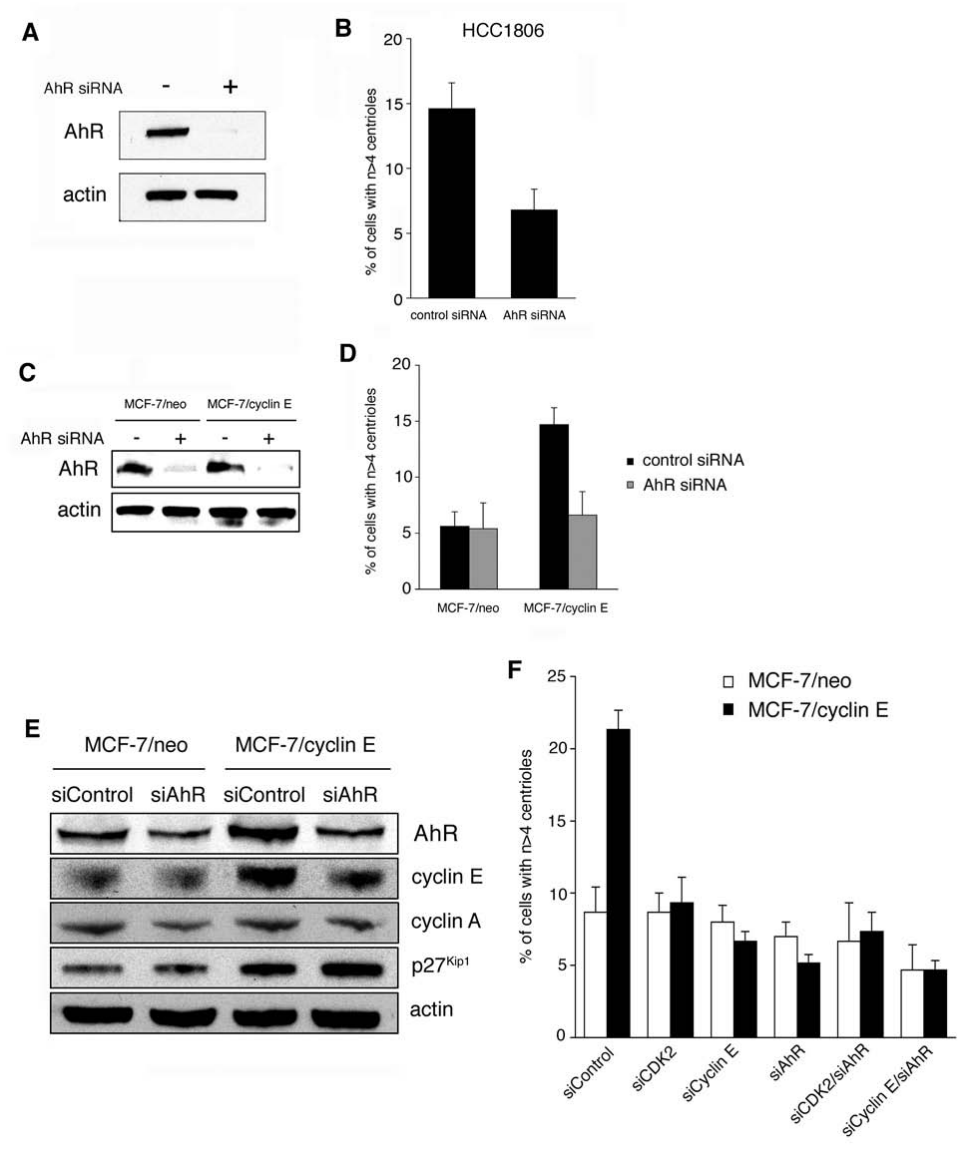
**Knock-down of the AhR reduces centriole aberrations in breast cancer cell lines**. (A, B) Immunoblot analysis of HCC1806 cells for AhR expression after transient transfection of cells with siRNA targeting the AhR or control siRNA duplexes. Immunoblot for Actin is shown to demonstrate protein loading (A). Quantification of the proportion of HCC1806 cells with aberrant centriole numbers following transfection with siRNA targeting the AhR or control siRNA. Each bar represents mean and standard error of at least three independent experiments with a minimum of 50 cells counted per experiment (B). (C, D) Immunoblot analysis of MCF-7/neo and MCF-7/cyclin E cells for AhR expression after transient transfection of cells with siRNA targeting the AhR or control siRNA duplexes. Immunoblot for actin is shown to demonstrate protein loading (C). Quantification of the proportion of cells with aberrant centriole numbers following transfection with siRNA targeting the AhR or control siRNA. Each bar represents mean and standard error of at least three independent experiments with a minimum of 50 cells counted per experiment (D). (E) Immunoblot analysis of MCF-7/neo and MCF-7/cyclin E cells following transient transfection (72 h) with either control siRNA (siControl) or siRNA targeting the AhR (siAhR). Immunoblots for AhR, cyclin E, cyclin A, p27^Kip1 ^and actin are shown. (F) Quantification of the proportion of MCF-7/neo cells (open bars) and MCF-7/cyclin E cells (black bars) with n > 4 centrioles following transient transfection with either control siRNA (siControl) or siRNA duplexes targeting CDK2 (siCDK2), cyclin E (siCyclin E), AhR (siAhR), CDK2 and AhR (siCDK2/siAhR) or cyclin E and AhR (siCyclin E/siAhR). Each bar represents mean and stardard error of two independent experiments each with triple quantification of at least 50 cells.

To further corroborate a correlation between the AhR and centrosome amplification, we next examined centriole overduplication in HCC1806 and MCF-7 cells following siRNA-mediated AhR depletion (Fig. 4). Knock-down of the AhR by siRNA (Fig. 4A) was associated with a significant 2.1-fold reduction of the proportion of HCC1806 cells with aberrant centriole numbers from 14.6% in controls to 6.8% in AhR-depleted cells (p ≤ 0.05; Fig. 4B).

Knock-down of the AhR in MCF-7/cyclin E cells (Fig. 4C) was likewise found to cause a statistically significant 2.2-fold reduction of the proportion of cells with aberrant centriole numbers from 14.7% in control siRNA-transfected MCF-7/cyclin E cells to 6.6% in AhR-depleted MCF-7/cyclin E cells (p ≤ 0.05; Fig. 4D). No significant change was detected in MCF-7/neo controls transfected with AhR siRNA in comparison to control siRNA (p > 0.05).

The finding that AhR knock-down in MCF-7/cyclin E cells reduced centriole overduplication to the baseline levels detected in MCF-7/neo control cells, but not below, raises the possibility that cyclin E and AhR may function in the same pathway. To explore this idea, we performed an immunoblot analysis of control siRNA- or AhR siRNA-transfected MCF-7/neo and MCF-7/cyclin E cells (Fig. 4E). We detected an increase of baseline AhR protein expression in MCF-7 overexpressing cyclin E (see siControl lane; Fig. 4E). On the other hand, knock-down of AhR expression led to a reduction of cyclin E protein expression in MCF-7/cyclin E cells (see siAhR lane; Fig. 4E). A modest decrease in cyclin A protein expression was seen in MCF-7/neo cells. MCF-7/cyclin E cells showed increased baseline levels of p27^Kip1 ^in comparison to MCF-7/neo cells but no major changes following AhR depletion were found. These results suggest a positive feedback mechanism between cyclin E and AhR and are in line with previous reports showing a pro-proliferative function of endogenous AhR in contrast to growth-suppressive activities of exogenously activated AhR.

To further support the notion that cyclin E and AhR may function in the same pathway, we performed an additional series of siRNA experiments (Fig. 4F). SiRNA-mediated knock-down of either CDK2, cyclin E or AhR alone or in combination was performed in MCF-7/neo or MCF-7/cyclin E cells. Knock-down of CDK2 alone led to a statistically significant 2.3-fold reduction of the proportion of cells with aberrant centriole numbers from 21.3% in control siRNA-transfected MCF-7/cyclin E cells to 9.3% (p ≤ 0.005). Knock-down of cyclin E alone as well as AhR alone also caused a statistically significant 3.2-fold and 4.1-fold reduction, respectively, to 6.7% (p ≤ 0.001) and 5.2% (p ≤ 0.005) in MCF-7/cyclin E cells. Co-depletion of CDK2 and AhR led to a statistically significant 2.9-fold reduction of the proportion of cells with aberrant centriole numbers to 7.3% (p ≤ 0.005) whereas co-depletion of cyclin E and AhR caused a statistically significant 4.5-fold reduction to 4.7% (p ≤ 0.001) in MCF-7/cyclin E cells.

Collectively, these findings demonstrate that continued endogenous AhR expression is critical for centriole overduplication and furthermore suggest that cyclin E and endogenous AhR may function in the same pathway.

### Overexpression of the AhR causes centriole overduplication

**Figure 5:**
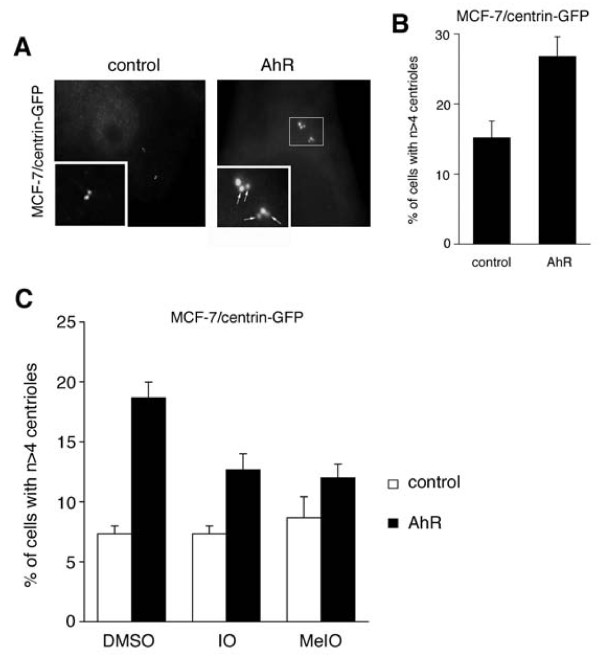
**Overexpression of the AhR stimulates centriole overduplication that can be reverted by indirubins**. (A) Fluorescence microscopic analysis of MCF-7 cells stably expressing centrin-GFP to visualize individual centrioles after transient transfection of cells with AhR or empty vector (control). Note the presence of two daughter centrioles at single maternal centrioles indicating aberrant daughter centriole synthesis (right panel). Arrows in right inset point to extra centrioles in an AhR-transfected cell. (B) Quantification of the proportion of MCF-7/centrin-GFP cells with aberrant centriole numbers following transfection with AhR or empty vector (control). Each bar represents mean and standard error of at least three independent experiments with a minimum of 50 cells counted per experiment. (C) Quantification of the proportion of MCF-7/centrin-GFP cells with n > 4 centrioles following transient transfection with either empty vector control (open bars) or AhR (black bars) and treatment with 0.1% DMSO, 1 μM IO or 1 μM MeIO starting 24 h after transfection of cells for an additional 24 h. Each bar represents mean and standard error of at least triple quantification of at least 50 cells.

To directly test whether overexpression of the AhR can disrupt centriole duplication control in breast cancers cells, MCF-7 cells manipulated to stably express centrin-GFP to visualize individual centrioles were transiently transfected with full-length human AhR (Fig. 5A). Ectopic expression of the AhR was found to stimulate aberrant centriole duplication with a statistically significant 1.8-fold increase from 15.2% in empty vector controls to 26.8% in AhR-transfected cells (p ≤ 0.05; Fig. 5B). A particularly striking phenotype that was detected in a proportion of MCF-7 cells was the formation of multiple daughter centrioles at single maternal centrioles following AhR overexpression (Fig. 5A; right panel). This phenotype is also referred to as centriole multiplication and indicates a genuine disruption of centriole duplication control.

We next tested whether cells in which centriole overduplication was induced by overexpression of the AhR would respond to the inhibitory effects of IO and MeIO. MCF-7/centrin-GFP cells were transiently transfected with empty vector or an AhR-encoding plasmid and treated with 1 μM IO or MeIO after 24 h for an additional 24 h (Fig. 5C). As expected, ectopic expression of the AhR led to a statistically significant 2.6-fold increase of cells with supernumerary centrioles from 7.3% in controls to 18.7% (p ≤ 0.001). Both, IO and MeIO caused a statistically significant reduction of cells with aberrant centriole numbers in AhR-transfected cells to 12.7% in IO-treated cells (p ≤ 0.05) and 12% in MeIO-treated cells (p ≤ 0.05).

These results further support a role of the AhR in promoting centriole overduplication in breast cancer cells and underscore that AhR agonists such as IO retain their ability to inhibit centriole overduplication even in cells with AhR overexpression.

## Discussion

Overexpression of the AhR has been detected in a number of cancerous and pre-cancerous lesions including breast and prostate cancer [2,4]. It is believed that the nuclear localization of the AhR in such lesions indicates its constitutive activation, although the precise molecular mechanisms leading to such activation remain elusive. Endogenous AhR that is not activated by exogenous ligand has, in general, pro-proliferative and tumor-promoting properties [1,8]. We report here a novel role of the AhR in centrosome duplication control. Given that supernumerary centrioles can cause cell division errors and chromosomal instability, this finding provides a potential link between the AhR and malignant progression. Remarkably, overexpression of the AhR can lead to centriole multiplication, a novel pathway of centriole overduplication that has only been found in the context of a few stimuli so far such as PLK4 overexpression or the human papillomavirus type 16 E7 oncoprotein (HPV-16 E7) [42,43].

Continued AhR expression was found to be necessary to promote centriole overduplication in HCC1806 breast cancer cells as well as MCF-7 cells stably expression cyclin E to hyperstimulate centriole overduplication. Ectopic cyclin E expression was associated with an upregulation of AhR protein expression while depletion of AhR in MCF-7 cells stably expressing cyclin E caused a decreased of cyclin E protein levels. Together with the finding that knock-down of either cyclin E or AhR has basically identical effects in terms of reduction of centriole overduplication, these results suggest that cyclin E and AhR may function in the same pathway to promote centriole overduplication. Since overexpression of cyclin E/CDK2 alone is necessary but not sufficient to induce centriole multiplication [30], it is likely that the AhR also affects additional components of the molecular circuitry that normally limits centriole biogenesis.

The AhR belongs to the bHLH family of transcription factors and two other members of this family, c-MYC and ID1, have been identified to also stimulate centrosome overduplication [30,44]. ID1 has been shown to interfere with a component of the ubiquitin-proteasome machinery to promote centrosome amplification [45]. Although the function of the AhR as part of a CUL4B-based E3 ubiquitin ligase [10,46] has not been explored in the absence of AhR ligand, it is possible that this activity is involved in centrosome overduplication, potentially through the degradation of a protein that normally restrains centriole biogenesis [42]. This notion is underscored by previous results showing that protein degradation plays a critical role in the regulation of daughter centriole biogenesis, in particular centriole multiplication [42], which we detected in a proportion of cells following ectopic expression of the AhR (Fig. 5). Although it remains to be experimentally confirmed, it is likely that the disruption of centriole duplication control by the AhR promotes centrosome-mediated cell division errors, chromosomal instability and malignant progression.

It is noteworthy that centrosome aberrations and multipolar mitoses are frequent findings in pre-cancerous lesions of the mammary gland and the prostate, both tumor entities in which aberrant AhR expression has been suggested to play a pathogenic role [2,4]. Our own results confirm and extend these results by showing that the coincidence of nuclear AhR overexpression and numerical centrosome aberrations correlates significantly with malignancy in mammary tissue samples. Surprisingly, aberrant AhR expression was detected not only in tumor samples but also in a considerable proportion of normal and hyperplastic mammary gland tissue specimens. Whether this reflects a role of endogenous AhR in normal cellular functions or a widespread pathological activation of this receptor by one or more ubiquitous xenobiotics remains to be determined. The finding that cellular alterations that are believed to be characteristic of malignancy can be detected in healthy individuals is not unprecedented. For example, hypermethylation of the p16^INK4A ^promoter has been reported in about 30% of tissue specimens from healthy women [47]. In any case, it will be important to investigate whether individuals with nuclear AhR overexpression in benign mammary tissue are at a higher risk to develop breast cancer.

There is an ongoing discussion whether the AhR can or should be exploited as a pharmacological target [48]. Results shown here provide compelling evidence that analogues of the *bis*-indole indirubin, which are known AhR agonists [31-33], can suppress centrosome overduplication in breast cancer cell lines *in vitro*. This activity was even detectable when centriole amplification was induced by AhR overexpression. We did not detect a significant inhibition of cell proliferation in cells treated with indirubins (data not shown), which is in line with our previous finding that centrosome overduplication can be inhibited independently from the cell division cycle [28]. Nonetheless, the activity spectrum of IO, which inhibits CDKs and has agonistic functions on the AhR, may be particularly favorable to target tumor cells. Since loss of genome stability is a progressive process, it will be necessary to target genomic instability at an early stage such as in pre-cancerous lesions. These lesions already frequently show signs of chromosomal instability such as centrosome aberrations, aneuploidy or DNA damage [49]. Based on these results, the development of suitable animal models to test indirubins as chemoprevention agents is clearly warranted.

## Conclusions

Our results support a role of endogenous AhR in promoting centrosome and centriole amplification in mammary tumors and breast cancer cell lines. However, AhR agonists such as indirubins were found to effectively suppress centriole overduplication even when stimulated by ectopic AhR expression. Our finding that a significant proportion of non-neoplastic breast tissue specimens showed nuclear overexpression of the AhR raises the question whether these individuals have an increased risk for centrosome-mediated cell division errors, aneuploidy and malignant progression. Collectively, these results provide a framework for future studies to manipulate the AhR for cancer chemoprevention.

## Competing interests

The authors declare that they have no competing interests.

## Authors' contributions

NK participated in performing the experiments, experimental design, data analysis and interpretation of the results. AD participated in the design of the experiments, data analysis and interpretation of the results. SM participated in performing the experiments and data analysis. PC participated in performing the experiments and data analysis. SD conceived the study, participated in performing the experiments, interpreted the results and wrote the manuscript. All authors have read and approved the final manuscript.

## Supplementary Material

Additional file 1 **AhR and centrosome staining results**. The data represent AhR and centrosome staining results in correlation to the histopathological diagnoses. Centrosome staining results were generated from three adjacent sections obtained from the same multi-tissue array (MTA). Click here for file

## References

[B1] Marlowe JL Puga A Aryl hydrocarbon receptor, cell cycle regulation, toxicity, and tumorigenesis J Cell Biochem 2005 96 1174 1184 10.1002/jcb.20656 16211578

[B2] Schlezinger JJ Liu D Farago M Seldin DC Belguise K Sonenshein GE Sherr DH A role for the aryl hydrocarbon receptor in mammary gland tumorigenesis Biol Chem 2006 387 1175 1187 10.1515/BC.2006.145 16972784

[B3] Koutros S Berndt SI Sinha R Ma X Chatterjee N Alavanja MC Zheng T Huang WY Hayes RB Cross AJ Xenobiotic metabolizing gene variants, dietary heterocyclic amine intake, and risk of prostate cancer Cancer Res 2009 69 1877 1884 10.1158/0008-5472.CAN-08-2447 19223546 PMC2662592

[B4] Gluschnaider U Hidas G Cojocaru G Yutkin V Ben-Neriah Y Pikarsky E beta-TrCP inhibition reduces prostate cancer cell growth via upregulation of the aryl hydrocarbon receptor PLoS One 5 e9060 10.1371/journal.pone.0009060 20140206 PMC2816705

[B5] Trombino AF Near RI Matulka RA Yang S Hafer LJ Toselli PA Kim DW Rogers AE Sonenshein GE Sherr DH Expression of the aryl hydrocarbon receptor/transcription factor (AhR) and AhR-regulated CYP1 gene transcripts in a rat model of mammary tumorigenesis Breast Cancer Res Treat 2000 63 117 131 10.1023/A:1006443104670 11097088

[B6] Yang X Solomon S Fraser LR Trombino AF Liu D Sonenshein GE Hestermann EV Sherr DH Constitutive regulation of CYP1B1 by the aryl hydrocarbon receptor (AhR) in pre-malignant and malignant mammary tissue J Cell Biochem 2008 104 402 417 10.1002/jcb.21630 18059014

[B7] Abdelrahim M Smith R Safe S Aryl hydrocarbon receptor gene silencing with small inhibitory RNA differentially modulates Ah-responsiveness in MCF-7 and HepG2 cancer cells Mol Pharmacol 2003 63 1373 1381 10.1124/mol.63.6.1373 12761348

[B8] Andersson P McGuire J Rubio C Gradin K Whitelaw ML Pettersson S Hanberg A Poellinger L A constitutively active dioxin/aryl hydrocarbon receptor induces stomach tumors Proc Natl Acad Sci USA 2002 99 9990 9995 10.1073/pnas.152706299 12107286 PMC126612

[B9] Puga A Barnes SJ Dalton TP Chang C Knudsen ES Maier MA Aromatic hydrocarbon receptor interaction with the retinoblastoma protein potentiates repression of E2F-dependent transcription and cell cycle arrest J Biol Chem 2000 275 2943 2950 10.1074/jbc.275.4.2943 10644764

[B10] Harper JW Chemical biology: a degrading solution to pollution Nature 2007 446 499 500 10.1038/446499a 17392771

[B11] Knockaert M Blondel M Bach S Leost M Elbi C Hager G Nagy SR Han D Denison M French M Indepedent actions on cyclin-dependent kinases and aryl hydrocarbon receptor mediate the antiproliferative effects of indirubins Oncogene 2004 23 4400 4412 10.1038/sj.onc.1207535 15077192

[B12] Lambert L Keyomarsi K Cell cycle deregulation in breast cancer: insurmountable chemoresistance or Achilles' heel? Adv Exp Med Biol 2007 608 52 69 full_text 17993232 10.1007/978-0-387-74039-3_4

[B13] Spruck CH Won KA Reed SI Deregulated cyclin E induces chromosome instability Nature 1999 401 297 300 10.1038/45836 10499591

[B14] Fukasawa K Oncogenes and tumour suppressors take on centrosomes Nat Rev Cancer 2007 7 911 924 10.1038/nrc2249 18004399

[B15] Duensing A Spardy N Chatterjee P Zheng L Parry J Cuevas R Korzeniewski N Duensing S Centrosome overduplication, chromosomal instability, and human papillomavirus oncoproteins Environ Mol Mutagen 2009 19326465 10.1002/em.20478

[B16] Azimzadeh J Bornens M Structure and duplication of the centrosome J Cell Sci 2007 120 2139 2142 10.1242/jcs.005231 17591686

[B17] Lingle WL Lukasiewicz K Salisbury JL Deregulation of the centrosome cycle and the origin of chromosomal instability in cancer Adv Exp Med Biol 2005 570 393 421 full_text 18727509 10.1007/1-4020-3764-3_14

[B18] Nigg EA Origins and consequences of centrosome aberrations in human cancers Int J Cancer 2006 119 2717 2723 10.1002/ijc.22245 17016823

[B19] Ganem NJ Godinho SA Pellman D A mechanism linking extra centrosomes to chromosomal instability Nature 2009 460 278 282 10.1038/nature08136 19506557 PMC2743290

[B20] Lingle WL Barrett SL Negron VC D'Assoro AB Boeneman K Liu W Whitehead CM Reynolds C Salisbury JL Centrosome amplification drives chromosomal instability in breast tumor development Proc Natl Acad Sci USA 2002 99 1978 1983 10.1073/pnas.032479999 11830638 PMC122305

[B21] Lingle WL Lutz WH Ingle JN Maihle NJ Salisbury JL Centrosome hypertrophy in human breast tumors: implications for genomic stability and cell polarity Proc Natl Acad Sci USA 1998 95 2950 2955 10.1073/pnas.95.6.2950 9501196 PMC19675

[B22] Lingle WL Salisbury JL Altered centrosome structure is associated with abnormal mitoses in human breast tumors Am J Pathol 1999 155 1941 1951 10595924 10.1016/S0002-9440(10)65513-7 PMC1866918

[B23] Duensing A Duensing S Guilt by association? p53 and the development of aneuploidy in cancer Biochem Biophys Res Commun 2005 331 694 700 10.1016/j.bbrc.2005.03.157 15865924

[B24] Duensing S A tentative classification of centrosome abnormalities in cancer Cell Biol Int 2005 29 352 359 10.1016/j.cellbi.2005.03.005 15890539

[B25] Parvin JD The BRCA1-dependent ubiquitin ligase, gamma-tubulin, and centrosomes Environ Mol Mutagen 2009 50 649 653 10.1002/em.20475 19274767

[B26] Li JJ Weroha SJ Lingle WL Papa D Salisbury JL Li SA Estrogen mediates Aurora-A overexpression, centrosome amplification, chromosomal instability, and breast cancer in female ACI rats Proc Natl Acad Sci USA 2004 101 18123 18128 10.1073/pnas.0408273101 15601761 PMC539804

[B27] Schneeweiss A Sinn HP Ehemann V Khbeis T Neben K Krause U Ho AD Bastert G Kramer A Centrosomal aberrations in primary invasive breast cancer are associated with nodal status and hormone receptor expression Int J Cancer 2003 107 346 352 10.1002/ijc.11408 14506732

[B28] Duensing S Duensing A Lee DC Edwards KM Piboonniyom S Manuel E Skaltsounis L Meijer L Munger K Cyclin-dependent kinase inhibitor indirubin-3'-oxime selectively inhibits human papillomavirus type 16 E7-induced numerical centrosome anomalies Oncogene 2004 23 8206 8215 10.1038/sj.onc.1208012 15378001

[B29] Shaye A Sahin A Hao Q Hunt K Keyomarsi K Bedrosian I Cyclin E deregulation is an early event in the development of breast cancer Breast Cancer Res Treat 2009 115 651 659 10.1007/s10549-008-0266-0 19107593 PMC6130890

[B30] Korzeniewski N Zheng L Cuevas R Parry J Chatterjee P Anderton B Duensing A Munger K Duensing S Cullin 1 functions as a centrosomal suppressor of centriole multiplication by regulating polo-like kinase 4 protein levels Cancer Res 2009 69 6668 6675 10.1158/0008-5472.CAN-09-1284 19679553 PMC2727667

[B31] Knockaert M Blondel M Bach S Leost M Elbi C Hager GL Nagy SR Han D Denison M Ffrench M Independent actions on cyclin-dependent kinases and aryl hydrocarbon receptor mediate the antiproliferative effects of indirubins Oncogene 2004 23 4400 4412 10.1038/sj.onc.1207535 15077192

[B32] Kawanishi M Sakamoto M Ito A Kishi K Yagi T Construction of reporter yeasts for mouse aryl hydrocarbon receptor ligand activity Mutat Res 2003 540 99 105 12972062 10.1016/s1383-5718(03)00174-8

[B33] Adachi J Mori Y Matsui S Takigami H Fujino J Kitagawa H Miller CA Kato T Saeki K Matsuda T Indirubin and indigo are potent aryl hydrocarbon receptor ligands present in human urine J Biol Chem 2001 276 31475 31478 10.1074/jbc.C100238200 11425848

[B34] Hinds PW Mittnacht S Dulic V Arnold A Reed SI Weinberg RA Regulation of retinoblastoma protein functions by ectopic expression of human cyclins Cell 1992 70 993 1006 10.1016/0092-8674(92)90249-C 1388095

[B35] Piel M Meyer P Khodjakov A Rieder CL Bornens M The respective contributions of the mother and daughter centrioles to centrosome activity and behavior in vertebrate cells J Cell Biol 2000 149 317 330 10.1083/jcb.149.2.317 10769025 PMC2175166

[B36] Kim DW Gazourian L Quadri SA Romieu-Mourez R Sherr DH Sonenshein GE The RelA NF-kappaB subunit and the aryl hydrocarbon receptor (AhR) cooperate to transactivate the c-myc promoter in mammary cells Oncogene 2000 19 5498 5506 10.1038/sj.onc.1203945 11114727

[B37] Duensing A Liu Y Spardy N Bartoli K Tseng M Kwon JA Teng X Duensing S RNA polymerase II transcription is required for human papillomavirus type 16 E7- and hydroxyurea-induced centriole overduplication Oncogene 2007 26 215 223 10.1038/sj.onc.1209782 16819507 PMC2228273

[B38] Duensing A Chin A Wang L Kuan SF Duensing S Analysis of centrosome overduplication in correlation to cell division errors in high-risk human papillomavirus (HPV)-associated anal neoplasms Virology 2008 372 157 164 10.1016/j.virol.2007.10.030 18036631 PMC2267749

[B39] Duensing A Liu Y Tseng M Malumbres M Barbacid M Duensing S Cyclin-dependent kinase 2 is dispensable for normal centrosome duplication but required for oncogene-induced centrosome overduplication Oncogene 2006 25 2943 2949 10.1038/sj.onc.1209310 16331279 PMC2225596

[B40] Duensing S Duensing A Crum CP Munger K Human papillomavirus type 16 E7 oncoprotein-induced abnormal centrosome synthesis is an early event in the evolving malignant phenotype Cancer Res 2001 61 2356 2360 11289095

[B41] Hoessel R Leclerc S Endicott JA Nobel MEM Lawrie A Tunnah P Leost M Damiens E Marie D Marko D Indirubin, the active constituent of a chinese antileukaemia medicine, inhibits cyclin-dependent kinases Nat Cell Biol 1999 1 60 67 10.1038/9035 10559866

[B42] Duensing A Liu Y Perdreau SA Kleylein-Sohn J Nigg EA Duensing S Centriole overduplication through the concurrent formation of multiple daughter centrioles at single maternal templates Oncogene 2007 26 6280 6288 10.1038/sj.onc.1210456 17438528 PMC2586811

[B43] Habedanck R Stierhof YD Wilkinson CJ Nigg EA The Polo kinase Plk4 functions in centriole duplication Nat Cell Biol 2005 7 1140 1146 10.1038/ncb1320 16244668

[B44] Hasskarl J Duensing S Manuel E Munger K The helix-loop-helix protein ID1 localizes to centrosomes and rapidly induces abnormal centrosome numbers Oncogene 2004 23 1930 1938 10.1038/sj.onc.1207310 14755252

[B45] Hasskarl J Mern DS Munger K Interference of the dominant negative helix-loop-helix protein ID1 with the proteasomal subunit S5A causes centrosomal abnormalities Oncogene 2008 27 1657 1664 10.1038/sj.onc.1210808 17891176

[B46] Ohtake F Baba A Takada I Okada M Iwasaki K Miki H Takahashi S Kouzmenko A Nohara K Chiba T Dioxin receptor is a ligand-dependent E3 ubiquitin ligase Nature 2007 446 562 566 10.1038/nature05683 17392787

[B47] Tlsty TD Crawford YG Holst CR Fordyce CA Zhang J McDermott K Kozakiewicz K Gauthier ML Genetic and epigenetic changes in mammary epithelial cells may mimic early events in carcinogenesis J Mammary Gland Biol Neoplasia 2004 9 263 274 10.1023/B:JOMG.0000048773.95897.5f 15557799

[B48] Zhang S Lei P Liu X Li X Walker K Kotha L Rowlands C Safe S The aryl hydrocarbon receptor as a target for estrogen receptor-negative breast cancer chemotherapy Endocr Relat Cancer 2009 16 835 844 10.1677/ERC-09-0054 19447902 PMC2766348

[B49] Duensing A Spardy N Chatterjee P Zheng L Parry J Cuevas R Korzeniewski N Duensing S Centrosome overduplication, chromosomal instability, and human papillomavirus oncoproteins Environ Mol Mutagen 2009 50 741 747 10.1002/em.20478 19326465

