# Supplementary material for: A novel role of the aryl hydrocarbon receptor (AhR) in centrosome amplification - implications for chemoprevention
Source: Mol Cancer. 2010 Jun 17;9:153. doi: 10.1186/1476-4598-9-153 (PMC2898706; doi:10.1186/1476-4598-9-153)
Supplement: Additional file 1 — AhR and centrosome staining results. The data represent AhR and centrosome staining results in correlation to the histopathological diagnoses. Centrosome staining results were generated from three adjacent sections obtained from the same multi-tissue array (MTA). [file 1476-4598-9-153-S1.DOC]

|  |  | **Centrosome (MTA I)** | **Centrosome (MTA II)** | **Centrosome (MTA III)** | **Centrosome** |
| --- | --- | --- | --- | --- | --- |
| **Tissue** | **AhR +/-** | **>2/1 or 2 (% abnormal)** | **>2/1 or 2 (% abnormal)** | **>2/1 or 2 (% abnormal)** | **Aberrations +/-** |
| NORMAL | - | 0/12 (0%) | NA | 0/8 (0%) | - |
| NORMAL | + | 0/8 (0%) | NA | 2/50 (3.8%) | + |
| NORMAL | + | 0/19 (0%) | NA | 0/44 (0%) | - |
| NORMAL | + | 0/100 (0%) | 0/50 (0%) | 0/50 (0%) | - |
| NORMAL | + | 1/100 (1%) | 1/50 (2%) | 0/60 (0%) | + |
| NORMAL | - | NA | NA | 0/5 (0%) | - |
| HYPERPLASIA | + | NA | NA | 0/53 (0%) | - |
| HYPERPLASIA | + | NA | NA | 2/77 (2.5%) | + |
| HYPERPLASIA | + | NA | NA | 0/50 (0%) | - |
| HYPERPLASIA | + | NA | NA | 0/50 (0%) | - |
| HYPERPLASIA | - | NA | 0/50 (0%) | NA | - |
| HYPERPLASIA | + | NA | 0/10 | 1/60 (1.6%) | + |
| HYPERPLASIA | + | NA | NA | 1/19 (5%) | + |
| HYPERPLASIA | + | NA | 0/50 (0%) | 0/57 (0%) | - |
| HYPERPLASIA | + | NA | 0/11 (0%) | 11/86 (11.3%) | + |
| HYPERPLASIA | - | 0/21 (0%) | NA | NA | - |
| HYPERPLASIA | - | 0/4 (0%) | 0/14 (0%) | NA | - |
| HYPERPLASIA | + | 1/52 (1.9%) | NA | 3/60 (4.8%) | + |
| HYPERPLASIA | + | 0/13 (0%) | NA | 1/30 (3.2%) | + |
| HYPERPLASIA | + | 0/40 (0%) | 0/25 (0%) | 3/31 (8.8%) | + |
| HYPERPLASIA | - | 1/40 (2.4%) | 1/20 (4.8%) | NA | + |
| HYPERPLASIA | + | 6/16 (27.3%) | NA | NA | + |
| DYSPLASIA | + | NA | 0/50 (0%) | 0/12 (0%) | - |
| DYSPLASIA | - | NA | 16/50 (24.2%) | NA | + |
| DYSPLASIA | + | NA | 9/50 (15.3%) | 3/29 (9.4%) | + |
| DYSPLASIA | + | 4/24 (14.3%) | 1/50 (2%) | NA | + |
| DYSPLASIA | + | NA | 1/46 (2.1%) | NA | + |
| DYSPLASIA | - | 1/51 (1.9%) | 0/20 (0%) | NA | + |
| DYSPLASIA | - | NA | 1/33 (2.9%) | NA | + |
| DYSPLASIA | - | 4/60 (6.3%) | 0/50 (0%) | NA | + |
| DYSPLASIA | + | 0/23 (0%) | 2/55 (3.6%) | NA | + |
| DYSPLASIA | + | 3/40 (7%) | 0/60 (0%) | NA | + |
| DYSPLASIA | + | NA | 2/50 (3.8%) | NA | + |
| DYSPLASIA | + | NA | 1/50 (2%) | NA | + |
| DYSPLASIA | + | 0/40 (0%) | 1/50 (2%) | NA | + |
| CANCER | - | 4/36 (10%) | 3/50 (5.7%) | NA | + |
| CANCER | + | NA | NA | 5/50 (9.1%) | + |
| CANCER | + | NA | NA | 3/34 (8.8%) | + |
| CANCER | + | 3/35 (7.9%) | 0/15 (0%) | NA | + |
| CANCER | + | NA | 1/28 (3.4%) | 2/50 (3.8%) | + |
| CANCER | + | NA | 1/28 (3.4%) | NA | + |
| CANCER | + | NA | 1/9 (10%) | NA | + |
| CANCER | + | NA | 2/45 (4.3%) | 4/30 (11.8%) | + |
| CANCER | + | NA | 0/8 (0%) | NA | - |
| CANCER | - | NA | NA | 4/50 (7.4%) | + |
| CANCER | + | NA | NA | 3/44 (6.4%) | + |
| CANCER | + | NA | 1/50 (2%) | 0/60 (0%) | + |
| CANCER | + | NA | NA | 6/31 (16.2%) | + |
| CANCER | + | NA | NA | 5/22 (18.5%) | + |
| CANCER | + | NA | 3/28 (9.7%) | 5/40 (11.1%) | + |
| CANCER | + | NA | NA | 3/43 (6.5%) | + |
| CANCER | + | NA | 2/50 (3.8%) | 5/36 (12.2%) | + |
| CANCER | + | NA | 2/50 (3.8%) | 6/70 (7.9%) | + |
| CANCER | + | NA | NA | 5/30 (14.3%) | + |
